# High-Resolution Maps of Mouse Reference Populations

**DOI:** 10.1534/g3.117.300188

**Published:** 2017-08-23

**Authors:** Petr Simecek, Jiri Forejt, Robert W. Williams, Toshihiko Shiroishi, Toyoyuki Takada, Lu Lu, Thomas E. Johnson, Beth Bennett, Christian F. Deschepper, Marie-Pier Scott-Boyer, Fernando Pardo-Manuel de Villena, Gary A. Churchill

**Affiliations:** * The Jackson Laboratory, Bar Harbor, Maine 04609; † Division Biotechnology and Biomedicine Centre of the Academy of Sciences, Institute of Molecular Genetics of the Academy of Sciences of the Czech Republic, Vestec, Prague 4, 142 20, Czech Republic; ‡ Department of Anatomy and Neurobiology, University of Tennessee Health Science Center, Memphis, Tennessee 38163; § National Institute of Genetics, Mishima, 411-8540, Japan; ** University of Colorado at Boulder, Colorado 80309; †† Institut de Recherches Cliniques, Montreal, Quebec, H2W 1R7, Canada; ‡‡ Institute for Behavioral Genetics, Department of Genetics, Lineberger Comprehensive Cancer Center, University of North Carolina at Chapel Hill, North Carolina 27599-7264

**Keywords:** chromosome substitution strains, recombinant inbred strains, mouse diversity genotyping array, gene conversions

## Abstract

Genetic reference panels are widely used to map complex, quantitative traits in model organisms. We have generated new high-resolution genetic maps of 259 mouse inbred strains from recombinant inbred strain panels (C57BL/6J × DBA/2J, ILS/IbgTejJ × ISS/IbgTejJ, and C57BL/6J × A/J) and chromosome substitution strain panels (C57BL/6J-Chr#<A/J>, C57BL/6J-Chr#<PWD/Ph>, and C57BL/6J-Chr#<MSM/Ms>). We genotyped all samples using the Affymetrix Mouse Diversity Array with an average intermarker spacing of 4.3 kb. The new genetic maps provide increased precision in the localization of recombination breakpoints compared to the previous maps. Although the strains were presumed to be fully inbred, we found residual heterozygosity in 40% of individual mice from five of the six panels. We also identified *de novo* deletions and duplications, in homozygous or heterozygous state, ranging in size from 21 kb to 8.4 Mb. Almost two-thirds (46 out of 76) of these deletions overlap exons of protein coding genes and may have phenotypic consequences. Twenty-nine putative gene conversions were identified in the chromosome substitution strains. We find that gene conversions are more likely to occur in regions where the homologous chromosomes are more similar. The raw genotyping data and genetic maps of these strain panels are available at http://churchill-lab.jax.org/website/MDA.

The laboratory mouse is the most widely used mammalian model organism for biomedical research. Among the key advantages of mice are a well-annotated reference genome (Chinwalla *et al.* 2002), over 100 strain-specific genome sequences (Keane *et al.* 2011; Morgan *et al.* 2016; Srivastava *et al.* 2017), and many genetic reference populations, including multi-parent strain panels (Collaborative Cross Consortium 2012) and outbred stocks (Churchill *et al.* 2012), and strains carrying null alleles at most protein coding genes. There are hundreds of readily available inbred strains that capture a wealth of genetic variants and display unique phenotypic characters (Beck *et al.* 2000; Yang *et al.* 2011).

Genetic reference populations of mice include collections of strains that reassort a fixed set of genetic variants such as chromosome substitution strain (CSS) and recombinant inbred strain (RIS) panels. CSS, also known as consomic strains, combine genomes of two founder inbred strains by substituting one chromosome pair from the *donor strain* into the genetic background of the *host strain* (Nadeau *et al.* 2012). The mouse genome is composed of 19 pairs of autosomal chromosomes, X and Y sex chromosomes, and a mitochondrial genome, thus a minimum of 22 strains could constitute a complete CSS panel. In some cases, it has proven difficult to introgress a specific entire donor strain chromosome into the host background, and the complete CSS panel may include partial chromosome substitutions and consists of >22 strains. RIS also combine genomes of two founder strains; they are derived from one or more generations of outcrossing followed by sibling mating to produce new inbred strains whose genomes are mosaics of the founder genomes (Williams *et al.* 2001). Both RIS and CSS panels have been successfully applied to the mapping of complex traits (Buchner and Nadeau 2015). RIS can be used in validation experiments by selecting a limited set (5–10) of strains that differ in haplotype in a region of interest to test for effects on phenotypes without the need of screening the whole set. They can be also used in follow up QTL studies involving the mouse strains present in a RIS panel (or strains that share the same haplotypes at the QTL).

We have carried out high-density genotyping of three RIS panels C57BL/6J × DBA/2J (BXD), ILS/IbgTejJ × ISS/IbgTejJ (LXS), C57BL/6J × A/J (AXB/BXA), and three CSS panels C57BL/6J-Chr#<A/J> (B6.A), C57BL/6J-Chr#<PWD/Ph> (B6.PWD), and C57BL/6J-Chr#<MSM/Ms> (B6.MSM) using the Affymetrix Mouse Diversity Array (MDA). The MDA includes ∼623,000 probe sets that assay single nucleotide polymorphisms (SNPs) plus an additional 916,000 invariant genomic probes targeted to genetic deletions or duplications (Yang *et al.* 2009). These data add value to the strain panels by more precisely localizing the recombination breakpoints between founder strains. In addition, they reveal some unexpected features in the genomes of individual strains.

## Materials and Methods

### Animals

We generated high-density genotype data for six mouse strain panels (Table 1): three panels of RIS and three panels of CSS. Mice for genotyping from five panels were available at the Jackson Laboratory (Bar Harbor, ME) or from BXD colony at University of Tennessee Health Science Center (UTHSC); DNA samples from the sixth panel, B6.MSM CSS, were provided by T. Shiroishi (National Institute of Genetics, Japan). Unless stated otherwise, we genotyped one mouse per strain. Most strains are represented by a single male animal (255 males) but, for four strains (BXD14, BXD54, BXD59, and BXD76), we genotyped an individual female. Samples were mainly from cases bred in 2008.

**Table 1:** Overview of the six panels: a type, founder strains, a number of strains, and a number of informative SNPs

Panel	Type	Founder Strains		# Strains	# Informative SNPs
AXB/BXA	RIS	C57BL/6J	A/J	25	101,397
LXS	RIS	ILS	ISS	64	79,808
BXD	RIS	C57BL/6J	DBA/2J	91	103,340
B6.A	CSS	C57BL/6J	A/J	22	101,397
B6.PWD	CSS	C57BL/6J	PWD/Ph	28	257,492
B6.MSM	CSS	C57BL/6J	MSM/Ms	29	231,441

The AXB/BXA RIS panel (Nesbitt and Skamene 1984) was derived from intercrosses of the C57BL/6J (B or B6) and A/J (A) strains. Note that, hereafter, the dam is denoted first and the sire last. Thus the difference between AXB and BXA strains is the direction of the intercross mating that generated (A×B)F1s or (B×A)F1s, respectively. We genotyped 25 strains: AXB strains 1, 2, 4–6, 8, 10, 12, 13, 15, 18, 23, and 24; and BXA strains 1, 2, 4, 11–14, 16, 17, and 24–26.

The LXS RIS panel (Williams *et al.* 2004) was generated at the Institute for Behavioral Genetics, Boulder, CO, from founder strains, Inbred Long-Sleep (L or ILS) and Inbred Short-Sleep (S or ISS). These founder strains were in turn derived as selection lines from a cross population with eight founder strains (A, AKR, BALB/c, C3H/Crgl/2, C57BL/Crgl, DBA/2, IS/Bi, and RIII). We genotyped 64 strains: LXS 3, 5, 7–9, 13, 14, 16, 19, 22–26, 28, 32, 34–36, 39, 41–43, 46, 48–52, 56, 60, 62, 64, 66, 70, 72, 73, 75, 76, 78, 80, 84, 86, 87, 89, 90, 92–94, 96–103, 107, 110, 112, 114, 115, 122, and 123.

The BXD RIS panel was derived from founder strains C57BL/6J (B or B6) and DBA/2J (D or D2) inbred mice in three epochs: epoch I, strains 1–32 (Taylor *et al.* 1975); epoch II, 33–42 (Taylor *et al.* 1999), and the epoch III advanced RIS 43–102 (Peirce *et al.* 2004). The latter were outcrossed for multiple generations before inbreeding. We genotyped 91 strains: BXD 1, 2, 5, 6, 8, 9, 11–16, 18–25, 27–36, 38–40, 42–45, 47–56, 59–71, and 73–102 (note that the designation of several BXD strains have been modified as a result of the genotyping results described in the present study, and BXD103 is now known as BXD73b).

The B6.A CSS panel (Nadeau *et al.* 2000) consists of 22 strains derived from C57BL/6J (host) and A/J (donor) by J. Nadeau at Case Western Reserve University. The panel includes 19 autosomes, X and Y chromosomes, and the mitochondrial genome.

The B6.PWD CSS panel (Gregorova *et al.* 2008) consists of 28 strains derived from C57BL/6J (host) and PWD/Ph (donor) by J. Forejt at the Institute of Molecular Genetics AS CR in Prague, Czech Republic, covering all chromosomes and the mitochondrial genome. To improve reproductive fitness, chromosomes 10, 11, and X were split between three strains each carrying either the proximal (p), middle (m), or distal (d) portion of the respective chromosome.

The B6.MSM CSS panel (Takada *et al.* 2008) consists of 29 strains derived from C57BL/6J (host) and MSM/Ms (donor) by T. Shiroishi at National Institute of Genetics in Mishima, Japan covering all chromosomes. Chromosomes 2, 6, 7, 12, 13, and X were split between two strains, each carrying either the centromeric (C) or telomeric (T) portion of the respective chromosome.

### Genotyping

DNA samples were prepared at the University of North Carolina according to the standard Affymetrix protocol, and were hybridized on the Affymetrix MDA at the Jackson Laboratory as described previously (Yang *et al.* 2009; Didion *et al.* 2012). The MDA probes (NCBI37/mm9) were mapped to genomic positions in GRCM38/mm10 assembly. CEL files and updated mapping information are available at ftp://ftp.jax.org/petrs/MDA/raw_data/. We used the R software package MouseDivGeno (Didion *et al.* 2012) to extract intensities from CEL files, but; for the purposes of this study, we developed a genotyping method that is based on the direct comparison of SNP probeset intensities between the sample and the founder strains of the corresponding panel. We selected the informative SNPs with intensity differences between founder strains for each panel (101,397 SNPs for AXB/BXA, 79,808 for LXS, and 103,340 for BXD). Both selection of informative SNPs and SNP calls were probeset intensity based. For each strain and each SNP, the call can be either A (if the signal is close to the first founder), B (if the signal is close to the second founder), or N to represent “notA/notB.” We note that the N category includes both no-call and heterozygous genotypes, and simply indicates that the intensity signal of the sample is far from both founder strains.

### Founder haplotype blocks

In order to define the haplotype blocks of founder genotypes with allowance for errors in individual SNP level genotype calls, we applied the Viterbi algorithm to smooth the genotyping. We used software implemented in the Hidden Markov Model (HMM) R package (Himmelmann 2010). We call the Viterbi algorithm iteratively: at each iteration we reestimated the HMM transition probabilities based on the Viterbi reconstruction of haplotype blocks. The iterations are repeated until we reach the convergence (Juang and Rabiner 1990).

Genetic maps computed from RIS panels consist of intervals assigned to one of the founders, and gaps that delimit the interval within which the inferred recombination event(s) have occurred. We refer to the latter as “recombination intervals.”

For RIS panels, we compared our maps to those available at http://www.genenetwork.org. GeneNetwork.org provides two genotype files for the BXDs: a “classic” set (pre-2017) of genotypes that have been used in most mapping studies since 2005 (Shifman *et al.* 2006), and new consensus genotypes (2017) that include updated data for BXD43 through BXD220 that were collected November 2015 and processed using the GigaMUGA array (Morgan *et al.* 2016). In the current study, we have compared MDA genotypes to the classic genotypes used through the end of 2016.

### Strain contamination

An RIS or CSS is considered to be contaminated if it carries a segment of genome that did not originate from one of the two founder strains. We developed an HMM to search for contamination. In contrast to our previous HMM analysis, here we select SNPs that were not informative (both founders have the same signal). In a contaminated region, the signal of a given strain is expected to contain a higher proportion of SNPs that differ from both founder strains. To reduce the false positive rate, only intervals covering three or more noninformative SNPs were reported.

### Copy number variants

To determine if any of the RIS or CSS strains carried copy number variations (CNVs) that differed from the copy number in the founder strains, we applied the *simpleCNV* function of the MouseDivGeno package (Didion *et al.* 2012). We accepted only those candidate CNV detections that had length >20 kb and covered ≤10 invariant genomic probes with *t*-statistic >5 (*P* < 1E−6).

### Gene conversions

Gene conversions are short tracts (<1 kb) of nonreciprocal transfer of genetic information between two homologs that occur during meiosis. In the case of RIS, it is difficult to distinguish gene conversion events from short haplotype blocks that are due to closely spaced recombination events that occurred in different meiosis. Therefore, we restricted our attention to the CSS panels. We searched for single or small groups of adjacent SNPs that derive from the host genotype, but occur on the donor chromosomes. We examined individual SNP intensities to identify those that are clearly derived from the host strain and are present in a region of donor strain haplotype.

### Sister strains

In a typical RIS panel, the lineages that give rise to each RIS are independent, and thus there should be no sharing of recombination events between strains. BXD strains from epoch III are an exception because they may share recombinations that arose in the outbreeding generations (Peirce *et al.* 2004). Therefore, we excluded these strains from this analysis. We detected excess sharing of recombination junctions (Z-score >5.0), as an indicator that two strains are more similar than expected by chance.

### Data availability

The authors state that all data necessary for confirming the conclusions presented in the article are represented fully within the article. The raw genotyping data and genetic maps of these strain panels are available at http://churchill-lab.jax.org/website/MDA.

## Results

### Global genotyping error

Global genotyping error—defined as a percentage of informative SNPs discordant with the haplotype assignment—is typically <1%, but is higher for haplotype blocks of *M. m. musculus* (PWD) and *M. m. molossinus* (MSM) origin than for *M. m. domesticus* blocks (B6, A, and D2) (Supplemental Material, Figure S1; the legends to supplementary figures are provided in File S1). This is likely to be caused by polymorphisms in, or near, the oligonucleotide probe sequence or its flanking restriction sites (Didion *et al.* 2012). There are a few outlying strains with a higher error rate than other strains from the same panel [AXB1, BXD15, BXD25, BXD85, BXD65a (formerly known as BXD92), BXD93, B6.A#Chr7, and B6.A#Chr10] likely due to low DNA quality or to processing of arrays.

### Residual heterozygosity

Residual heterozygosity is present in some strains from each panel, except for the AXB/BXA strains, which appear to be fully inbred (Table 2). The detected heterozygous regions are an underestimate of percentage of segregating variation that is present in each strain because only a single animal per strain was genotyped. The presence of heterozygous strains in large RIS panels is not surprising. We estimated that, in the absence of selection, an RIS strain needs, on average, 24 generations of sib-mating to reach a heterozygosity rate <1%, and 36 generations to reach complete fixation. However, there is a significant variation in the number of generations required to achieve these landmarks (Broman 2005). For a panel of 22 strains (the size of a full CSS panel), 53 generations are required, on average, to achieve complete fixation for all its strains in the absence of selection.

**Table 2:** Residual heterozygosity and CNV (deletion/extra copy) in the six panels

Panel	Number of Strains	# Strains with Heterozygous Segment	%	# Strains with Deletion	%	# Strains with Extra Copy	%
AXB	25	0	0	5	20	1	4
LXS	64	35	55	12	19	1	2
BXD, Epoch I	26	0	0	15	58	6	23
BXD, Epoch II	8	3	38	1	13	1	13
BXD, Epoch III	57	34	60	7	12	2	4
B6.A	22	3	14	2	9	2	9
B6.PWD	28	9	32	0	0	0	0
B6.MSM	29	2	7	12	41	1	3

### De novo deletions and duplications

We detected 64 *de novo* deletions and 14 *de novo* duplications, with lengths ranging from 21 kb to 8.4 Mb, affecting 111 Ensembl genes (Table S1). Table 2 summarizes the frequency of strains with heterozygosity, deletions, and duplications. We observe that a longer time of inbreeding is associated with lower heterozygosity but more structural changes. This is seen most clearly by comparing different epochs of the BXD panel.

#### High-density genotyping identifies unexpected haplotype blocks in CSS panels:

We observe 27 haplotype blocks from the host strain in the proximal or distal regions of the donor chromosome across the three CSS panels (Table 3). These events are undesirable but not unexpected due to the distribution of markers used for CSS development (Nadeau *et al.* 2000). We also observe strains in which a host haplotype block occurs in the middle of an introgressed donor chromosome or a donor haplotype block occurs in a host chromosome. We observed seven such events distributed across all three CSS panels (bold face highlights in Table 3).

**Table 3:** Unexpected haplotype blocks in all CSS panels

Panel	Strain	Chr.	Start	End	Length	Should Be	Actually Is	# Ensembl Genes
A.B6	C57BL/6J-Chr1A/J/NaJ	1	3,211,051	22,830,804	19,619,754	A	B6	117
A.B6	C57BL/6J-Chr1A/J/NaJ	1	192,442,075	195,365,691	2,923,617	A	B6	25
A.B6	C57BL/6J-Chr4A/J/NaJ	4	154,799,715	156,166,747	1,367,033	A or B6	Het	63
A.B6	C57BL/6J-Chr5A/J/NaJ	5	149,410,906	150,567,049	1,156,144	A or B6	Het	16
**A.B6**	**C57BL/6J-Chr8A/J/NaJ**	**11**	**36,650,633**	**42,751,289**	**6,100,657**	**B6**	**A**	28
A.B6	C57BL/6J-Chr10A/J/NaJ	10	127,645,772	129,615,258	1,969,487	A or B6	Het	102
A.B6	C57BL/6J-Chr16A/J/NaJ	16	93,670,025	98,040,454	4,370,430	A	B6	47
A.B6	C57BL/6J-Chr17A/J/NaJ	17	3,071,428	6,154,773	3,083,346	A	B6	25
**PWD.B6**	**C57BL/6J-Chr1PWD/ForeJ**	**3**	**123,275,916**	**143,575,204**	**20,299,289**	**B6**	**Het**	138
PWD.B6	C57BL/6J-Chr3PWD/ForeJ	3	24,121,111	24,179,212	58,102	PWD	B6	1
**PWD.B6**	**C57BL/6J-Chr4PWD/ForeJ**	**5**	**148,956,085**	**151,725,288**	**2,769,204**	**B6**	**Het**	33
PWD.B6	C57BL/6J-Chr9PWD/ForeJ	9	123,944,659	124,087,880	143,222	PWD	B6	3
PWD.B6	C57BL/6J-Chr10.1PWD/ForeJ	10	57,607,018	60,613,285	3,006,268	PWD or B6	Het	37
PWD.B6	C57BL/6J-Chr10.2PWD/ForeJ	10	45,150,578	51,959,138	6,808,561	PWD or B6	Het	21
PWD.B6	C57BL/6J-Chr10.2PWD/ForeJ	10	95,379,265	101,638,084	6,258,820	PWD or B6	Het	32
PWD.B6	C57BL/6J-Chr10.3PWD/ForeJ	10	73,546,548	74,465,198	918,651	PWD or B6	Het	1
PWD.B6	C57BL/6J-Chr11.1PWD/ForeJ	11	3,105,931	3,877,120	771,190	PWD or B6	Het	29
PWD.B6	C57BL/6J-Chr11.1PWD/ForeJ	11	79,051,423	79,574,667	523,245	PWD or B6	Het	10
PWD.B6	C57BL/6J-Chr11.2PWD/ForeJ	11	35,418,368	43,961,733	8,543,366	PWD or B6	Het	53
PWD.B6	C57BL/6J-Chr11.3PWD/ForeJ	11	120,588,649	121,967,849	1,379,201	PWD	B6	60
PWD.B6	C57BL/6J-Chr12PWD/ForeJ	12	116,831,193	120,014,765	3,183,573	PWD	B6	16
PWD.B6	C57BL/6J-Chr19PWD/ForeJ	19	60,070,470	61,261,300	1,190,831	PWD	B6	20
PWD.B6	C57BL/6J-ChrX.3PWD/ForeJ	X	167,416,568	169,593,020	2,176,453	PWD	B6	16
MSM.B6	C57BL/6J-Chr4-MSM	4	24,485,868	24,671,707	185,840	MSM	B6	3
MSM.B6	C57BL/6J-Chr6C-MSM	6	3,180,317	3,410,126	229,810	MSM	B6	2
MSM.B6	C57BL/6J-Chr6T-MSM	6	147,160,651	149,556,829	2,396,179	MSM	B6	39
MSM.B6	C57BL/6J-Chr11-MSM	11	121,160,079	121,967,849	807,771	MSM	B6	21
**MSM.B6**	**C57BL/6J-Chr12C-MSM**	**1**	**195,151,543**	**195,285,766**	**134,224**	**B6**	**Het**	2
**MSM.B6**	**C57BL/6J-Chr13T-MSM**	**18**	**23,532,046**	**26,528,643**	**2,996,598**	**B6**	**Het**	24
MSM.B6	C57BL/6J-Chr14-MSM	14	122,545,401	124,751,019	2,205,619	MSM	B6	10
MSM.B6	C57BL/6J-Chr15-MSM	15	102,415,461	103,628,026	1,212,566	MSM	B6	46
MSM.B6	C57BL/6J-Chr16-MSM	16	95,378,122	98,069,653	2,691,532	MSM	B6	28
MSM.B6	C57BL/6J-Chr19-MSM	19	57,068,415	60,681,568	3,613,154	MSM	B6	27

#### High-density genotyping improves map accuracy in RIS panels:

To validate our haplotype assignment, and to estimate the level of improvement, we compared our maps to the versions available at www.genenetwork.org (LXS and BXD) or provided by Institut de recherches cliniques de Montréal (AXB/BXA). There was a high concordance (99.8% LXS, 98.1% BXD, and 99.5% ABX/BXA) between new and old maps for intervals that were assigned to one of the founder in both maps. The new maps decreased the level of uncertainty, measured as the sum of length of recombination intervals by 66% in the AXB/BXA panel, 41% in the BXD panel, and 5% in the LXS panel. This improvement mirrors the increase in the number of informative markers: from 792 to 101,397 (AXB/BXA), from 3796 to 103,341 (BXD), and from 2649 to 79,808 (LXS), respectively (Figure S2).

##### Strain contamination in the AXB/BXA panel:

An unexpected observation in AXB/BXA RIS panel, was the presence of six intervals that are not derived from either A or B6 inbred strains. Three chromosomes (chr14, chr15, and chr16) of the AXB1 strain, two chromosomes (chr5 and chr16) of AXB2, and one chromosome (chr13) of BXA1 are affected by contamination. Based on comparison to genotypes from a large panel of inbred strains (Yang *et al.* 2011), we conclude that the contamination derived from a strain that is closely related to DBA/2J.

##### Recombination rate:

**Figure 1:**
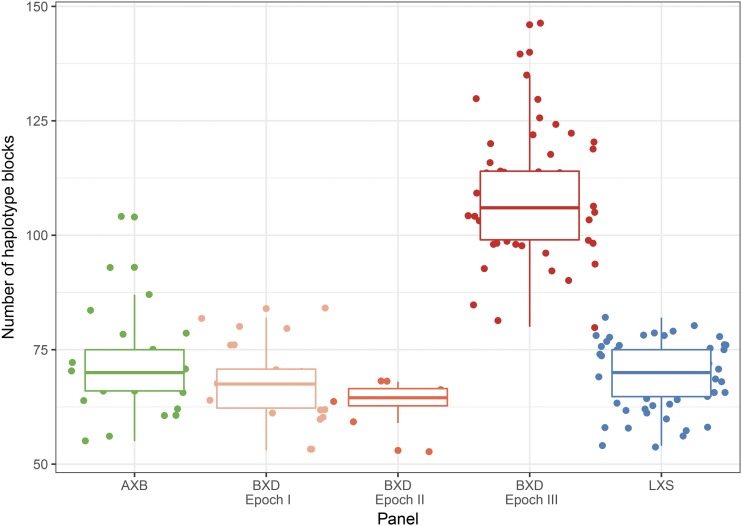
Number of founder haplotype blocks in RIS panels. The number of founder blocks for each strain is indicated as a point, with jitter for clarity. The boxplot indicates median and quartiles of each panel. Results for the BXD panel are broken down by three breeding epochs (I, II, and III); the increased number of recombination event in epoch III reflects additional generation of outbreeding used in the derivation of these strains.

The distribution of the number of recombination events is similar across all panels (see Figure 1 and Table S2) with the exception of the advanced RIS BXD (epoch III), which has more recombination events per chromosome due to additional generations of outbreeding. The number of recombination events per strain ranges from 32 (BXD32) to 84 (BXA17) among the classical RIS, and from 60 (BXD53) to 127 (BXD47) among the advanced BXD panel. These numbers of recombination events fall within the 95% prediction interval from simulations (using Python code from Welsh and McMillan 2012).

Most recombination events in the RIS panels are unique, but some recombination intervals overlap and could result from independent recurrent events or from shared ancestry between RIS during the inbreeding process. The most frequently shared recombination event occurs in 8 out of 25 samples of the AXB RIS panel (Chr10: 66,730,215–67,348,211). Moreover, in seven out of eight cases (*P* = 0.07), the polarity of the event is in the same direction: from B6 segment (proximal: 66,730,214 bp) to A/J segment (distal: 67,348,212 bp). Additional shared recombination intervals are listed in Table S3, and the recombination frequency is visualized in Figure S3. Higher recombination rates observed in the distal region of chromosomes are expected (Liu *et al.* 2014).

##### Sister strains:

**Figure 2:**
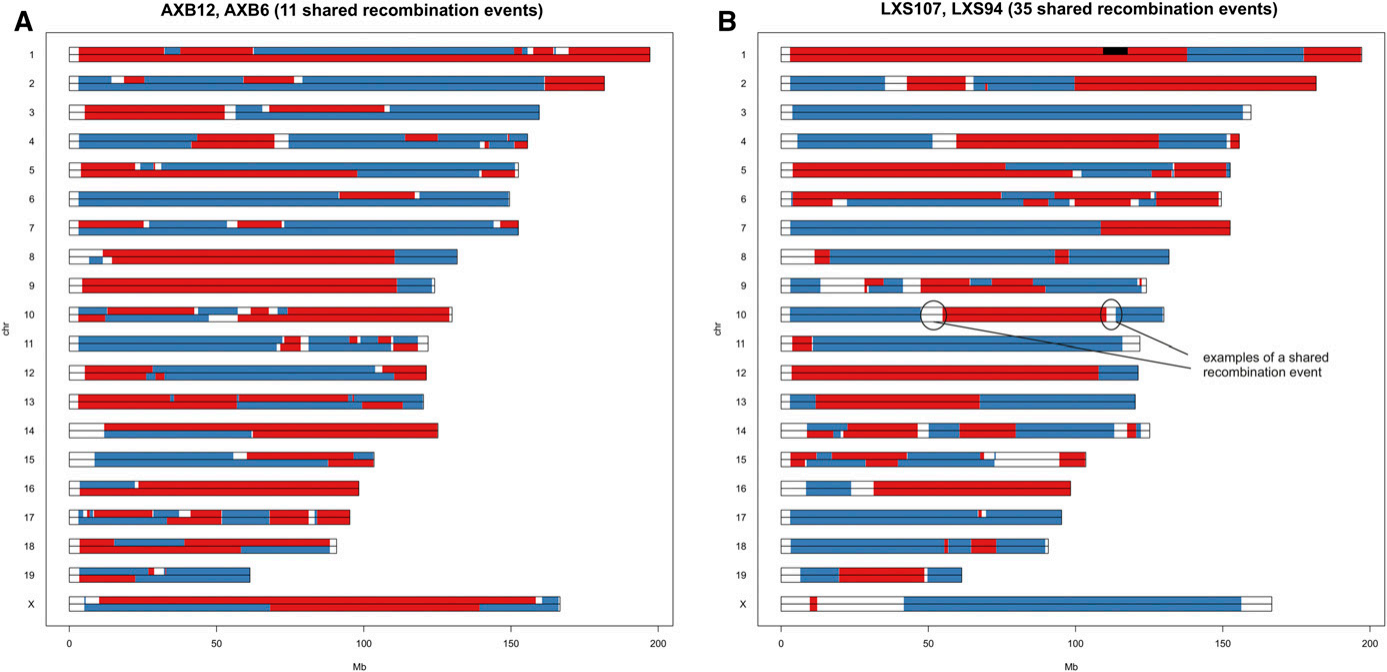
Sister strains in RIS panels. Side-by-side comparison of sister strains AXB6 *vs.* AXB12 (red, B6; blue, A) and LXS94 *vs.* LXS107 (red, L; blue, S) illustrates the extent of shared haplotype blocks.

Sister strains are strains related by descent from incompletely inbred ancestors during the breeding process. They can be identified because they share a large number of recombination intervals with the same proximal to distal polarity of founder haplotypes. Not surprisingly, most of the sister strains are detected for the advanced BXD panel (six pairs + six larger groups, totally comprising 40 strains). However, two pairs of strains are present in the AXB and LXS panels: AXB6–AXB12 and LXS94–LXS107. These strains share more recombination intervals with the same founder strain polarity than expected by chance (Figure 2).

##### The MDA array detects short gene conversions in CSS panels:

**Figure 3:**
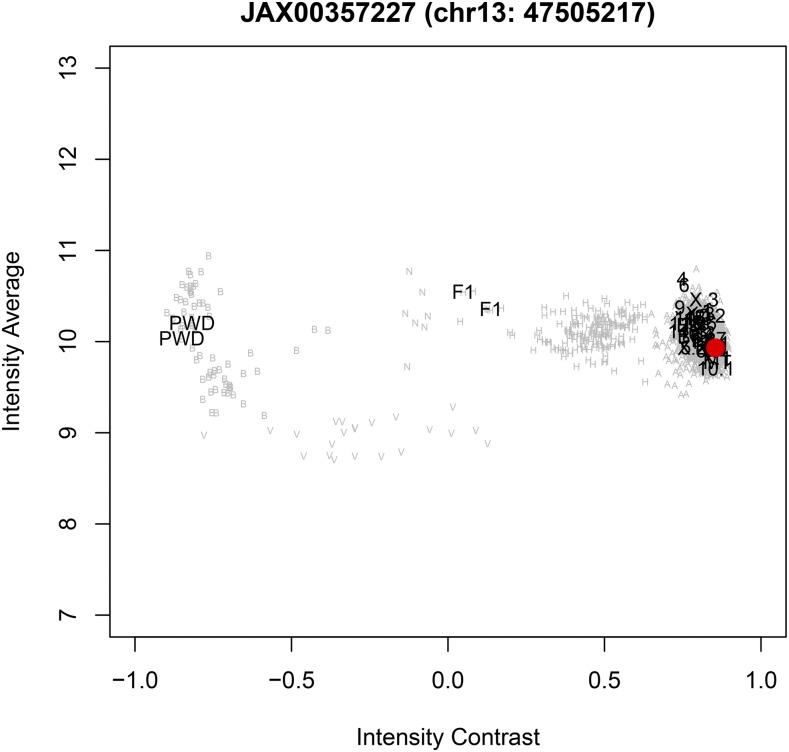
Gene conversion in a CSS strain. Strain B6.PWD13 has an unexpected founder genotype at marker JAX00357227 marker (Chr 13: 47,505,217 bp). Average and contrast signal intensities are plotted for all B6.PWD strains. Numbers indicate the CSS strains by substituted chromosome with B6. PWD13 is highlighted by the red circle. Also indicated on the plot are founder strains B6, and PWD and their F1 hybrids. The B.PWD13 data should be similar to PWD but is actually close to B6, indicating a putative gene conversion. Gray letters indicate genotype calls for 1902 additional samples in the MDA database (A, B6 allele homozygous; B, PWD allele homozygous; H, heterozygous; V, vino; N, no call).

We searched for putative gene conversions in the introgressed donor chromosomes of CSS panels. We identified small regions typically spanning just one informative SNP that have genotypes consistent with the host strain instead of the donor strain (Figure 3). In total, we identified 28 putative gene conversions: 17 in the B6.A CSS panel, seven in the B6.PWD CSS panel, and four in the B6.MSM CSS panel (Table 4).

**Table 4:** Short gene conversions in CSS panels

Panel	snp Id	Chr	Position	Allele A	Allele B	rs Number	Gene Symbol	Function Class
B6.A	JAX00254769	1	72747910	C	T	rs50360495	N/A	Intergenic
B6.A	JAX00506852	2	148712373	G	T	rs28225187	Napb	Intron
B6.A	JAX00517779	3	28468788	G	A	rs29689086	Tnik	Intron
B6.A	JAX00518655	3	31991151	G	A	rs49710262	N/A	Intergenic
B6.A	JAX00544220	4	7146585	C	T	rs27658062	N/A	Intergenic
B6.A	JAX00548886	4	41108534	C	T	rs27765251	N/A	Intergenic
B6.A	JAX00589927	5	101080632	A	G	rs31987722	N/A	Intergenic
B6.A	JAX00630284	6	146284916	C	A	rs30468531	Itpr2	Intron
B6.A	JAX00154063	7	89592185	G	A	rs51617084	N/A	Intergenic
B6.A	JAX00015582	10	20181498	G	C	rs29339980	Mtap7	Intron
B6.A	JAX00290764	10	62127533	C	T	rs46386144	N/A	Intergenic
B6.A	JAX00297554	10	103149924	A	G	rs47130688	Lrriq1	Intron
B6.A	JAX00306860	11	30181044	G	A	rs26860826	Spnb2	Intron
B6.A	JAX00364408	13	81444533	G	A	rs29225071	Gpr98	Exon (coding nonsynonymous)
B6.A	JAX00065772	15	102500631	T	C	rs13482749	Map3k12	Exon (coding synonymous)
B6.A	JAX00431551	17	11319650	G	A	rs33634737	Park2	Intron
B6.A	JAX00439159	17	44034125	A	G	rs33551899	Rcan2	Intron
B6.PWD	JAX00486683	2	32,226,493	A	C	rs28259595	5830434P21Rik	Intron
B6.PWD	JAX00507172	2	150,590,002	C	T	rs27373039	2310001A20Rik	Intron
B6.PWD	JAX00171651	9	57,610,867	C	T	rs30230810	Lman1l	Intron
B6.PWD	JAX00708417	9	123,590,594	C	A	rs36948070	Sacm1l	Intron
B6.PWD	JAX00357227	13	47,409,848	T	C	rs47221967	N/A	Intergenic
B6.PWD	JAX00072010	16	85,470,815	A	G	rs50630491	Cyyr1	Intron
B6.PWD	JAX00477099	19	38,677,937	T	C	rs31075313	Plce1	Intron
B6.MSM	JAX00250951	1	53216029	G	T	rs32733914	Pms1	Intron
B6.MSM	JAX00526581	3	72819995	G	A	rs37284921	N/A	Intergenic
B6.MSM	JAX00599346	5	139952853	T	G	rs32296220	A930017N06Rik	Intron
B6.MSM	JAX00427113	16	87664971	C	A	rs47532274	N/A	Intergenic

##### Online access to genetics maps and MDA genotypes:

For easy access, we provide a compilation of Mouse Diversity Array data, annotation and supporting software at http://churchill-lab.jax.org/website/MDA. Resources to support our analysis of RIS and CSS strains include an online viewer, where maps can be viewed and downloaded either as a list of intervals or as CSV files ready to be imported to the R/qtl package (Broman and Sen 2009). Source code for the viewer is also available on Github, https://github.com/simecek/RIS-map-viewer. Researchers interested in comparing those reference populations to genotypes of other mouse strains processed on MDA arrays can use the MDA viewer. The entire database, consisting of 1902 MDA arrays, is available for download as SQLite database or as individual CEL files ftp://ftp.jax.org/petrs/MDA/.

## Discussion

We have characterized 180 RIS and 79 CSS strains from six popular and valuable resources, and provided online access to these data. These panels were developed at different times and genotyped with lower density sets of markers. High-density genotyping with the number of informative SNPs, ranging between 79,000 and 257,000, provide maps with higher resolution. In this study, we achieved a median spacing between informative markers 5.7 kb (AXB), 5.4 kb (BXD), 5.6 kb (LXS), 4.6 kb (B6.PWD), and 5.2 kb (B6.MSM), respectively. This enabled us to identify unusual features such as regions of residual heterozygosity, contamination by a nonfounder strain and *de novo* structural variants. These genotyping arrays are part of 1902 samples processed on MDA platform that can be accessed from http://churchill-lab.jax.org/website/MDA.

Genetic reference panels are valuable, in part, because of the ability of generate animals with identical genomes in the number and timespan dictated by the researcher. Replication increases the accuracy of phenotype measurements (Belknap 1998), and allows for integration of data over space, time, and environment. While it is convenient to think of all mice from an inbred strain as identical, we provide evidence that this view is not always warranted. Residual heterozygosity may be due to stochasticity in the inbreeding process, or it may reflect biological constraints that prevent full inbreeding of a strain. Genetic drift operates in each of these populations, and low-density genotyping in selected regions of the genome, leaves room for undesired or unexpected surprises. In a typical CSS strain the average proportion of the donor genome present in other chromosomes is expected to be 0.2% (Nadeau *et al.* 2000). Over our three CSS panels, the average length of unexpected genotype was 1.5 Mb. The length of intervals ranges (Table 3) from <1 Mb (one gene) to 20 Mb (138 genes).

For gene conversions, whole genome sequencing of CSS panels (and RIS) will likely reveal more examples and provide better estimates of converted regions and their length. However, our results suggest that gene conversions are more probable in regions where founders’ genomes are very similar. We observe significantly more conversions on the B6.A panel than in the other two CSS panels (17 *vs.* 7 and 4, Fisher exact test, *P* = 0.046), despite the fact that the number of informative markers is lower and therefore our ability to detect gene conversions reduced. Based on this result, we hypothesize that gene conversions occur preferably in regions of low sequence diversity between homologous chromosomes. If that is true, then they will have fewer genetic consequences due to lower chance to cause distinguishing polymorphism. Roughly, we estimate that 0.005% of the genome is affected by gene conversion (avg. # gene conversions/# informative SNPs = 28/3/200,000). The real number of gene conversions is likely to be higher because we were only able to identify gene conversions that overlap informative SNP probes in the array.

We found no evidence of allele frequency imbalance (one allele present with significantly higher frequency than the other allele in a RIS panel) that has been observed in other species (Taudt *et al.* 2016). Nor did we detect any epistatic selection between founder strains or alleles with different subspecies origin (permutation test, data not shown). This is in sharp contrast with mouse multiparent populations such as the Collaborative Cross and Diversity Outbred (Chesler *et al.* 2016; Shorter *et al.* 2017; Srivastava *et al.* 2017) in which both distorted allele frequencies and epistatic selection are common. Due to limited number of strains in mouse RI panels, we may have missed small distortions.

We observed an inverse relationship between residual heterozygosity and drift (Table 2). For a given panel, even 20 generations of inbreeding is not enough to fix all heterozygous regions. On the other hand, populations kept for many generations will accumulate SNPs, small indels, and structural variants in their genomes (Simecek *et al.* 2015; Srivastava *et al.* 2017). Strategies to reduce drift in breeding colonies have been developed, including the embryo cryopreservation program at The Jackson Laboratory (Taft *et al.* 2006). However, genetic drift can be also harnessed by geneticists to simplify and accelerate the identification of causal variants responsible for phenotypic differences between substrains (Srivastava *et al.* 2017). These so-called reduced complexity crosses are excellent examples of the potential benefits of genetic drift (Kumar *et al.* 2013).

## Supplementary Material

Supplemental material is available online at www.g3journal.org/lookup/suppl/doi:10.1534/g3.117.300188/-/DC1.
