# Supplementary figures and images for: High-Resolution Maps of Mouse Reference Populations
Source: G3 (Bethesda). 2017 Aug 23;7(10):3427–34. doi: 10.1534/g3.117.300188 (PMC5633391; doi:10.1534/g3.117.300188)

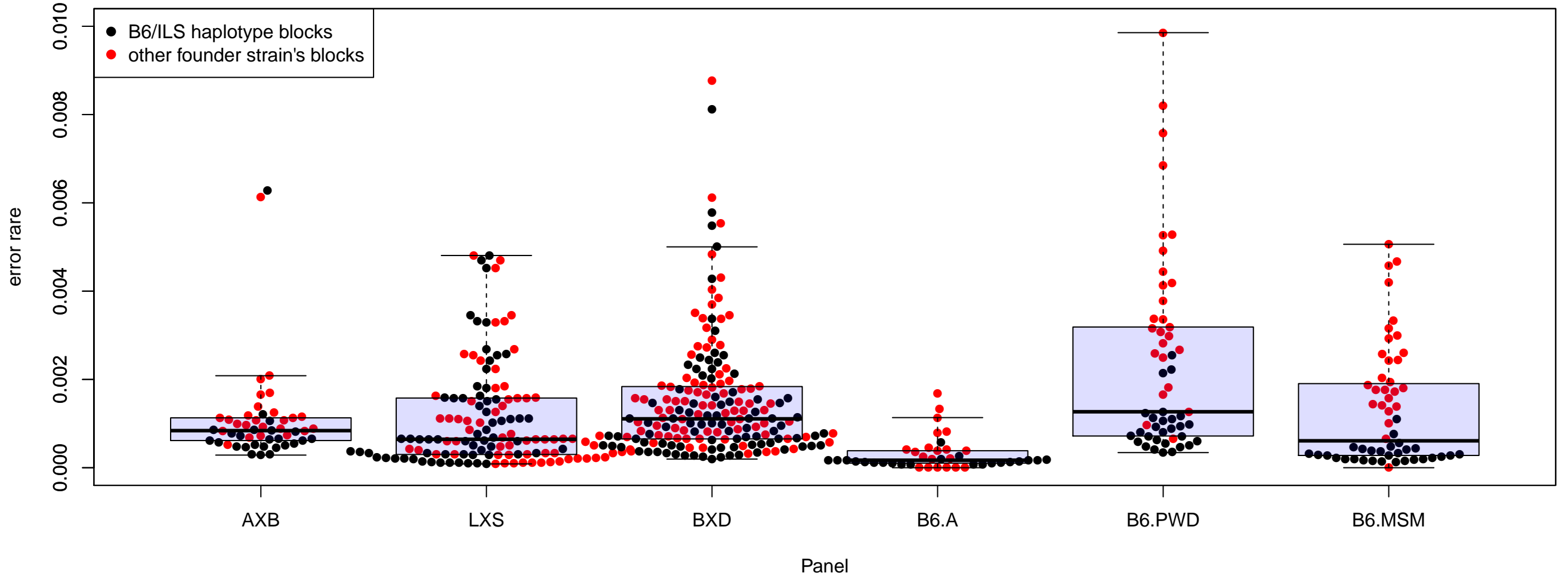

Supplement: Supplementary file 1 [file 3427FigureS1.pdf]

AXB / BXA

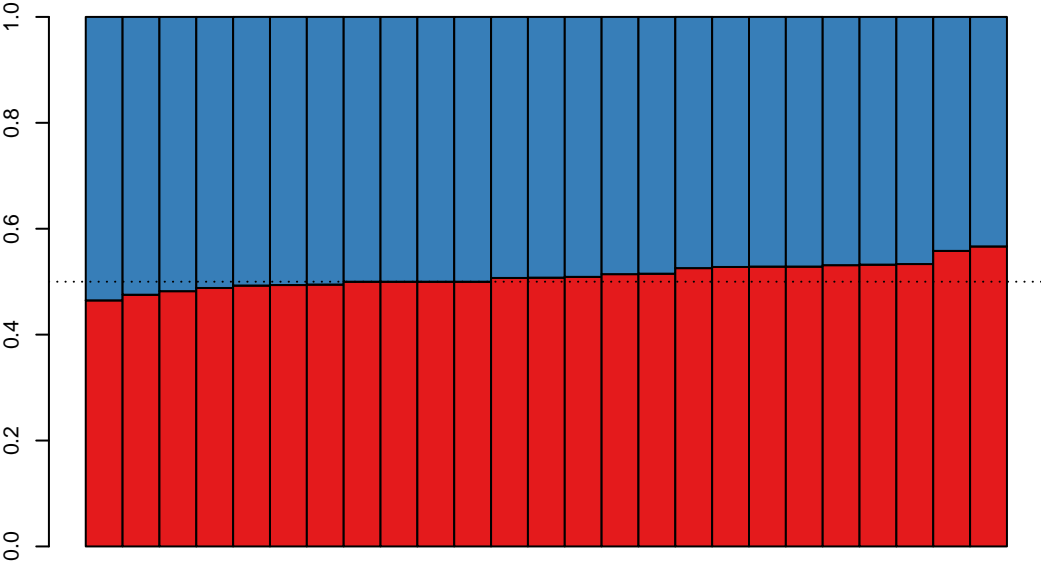

BXD

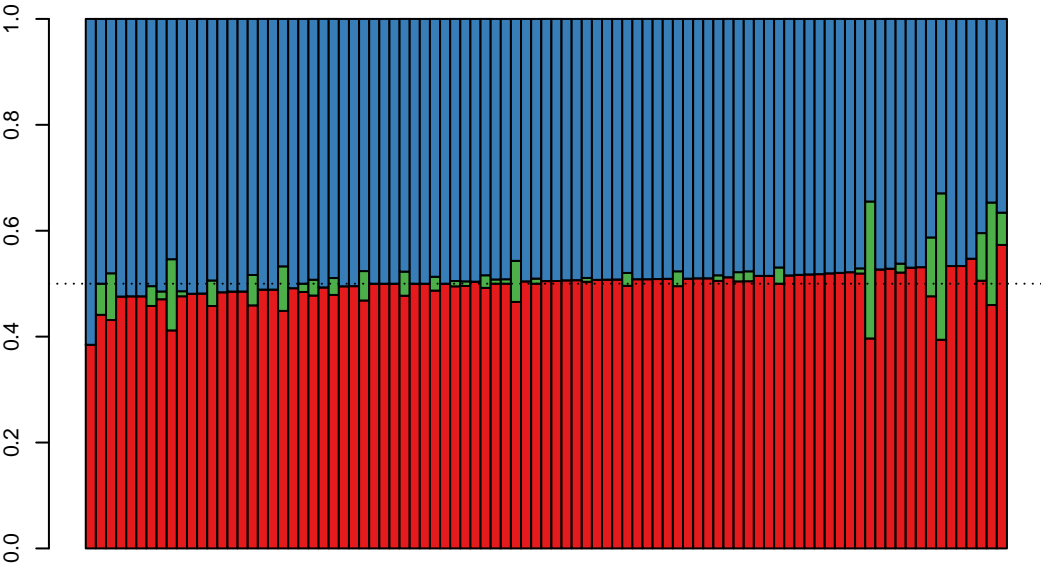

LXS

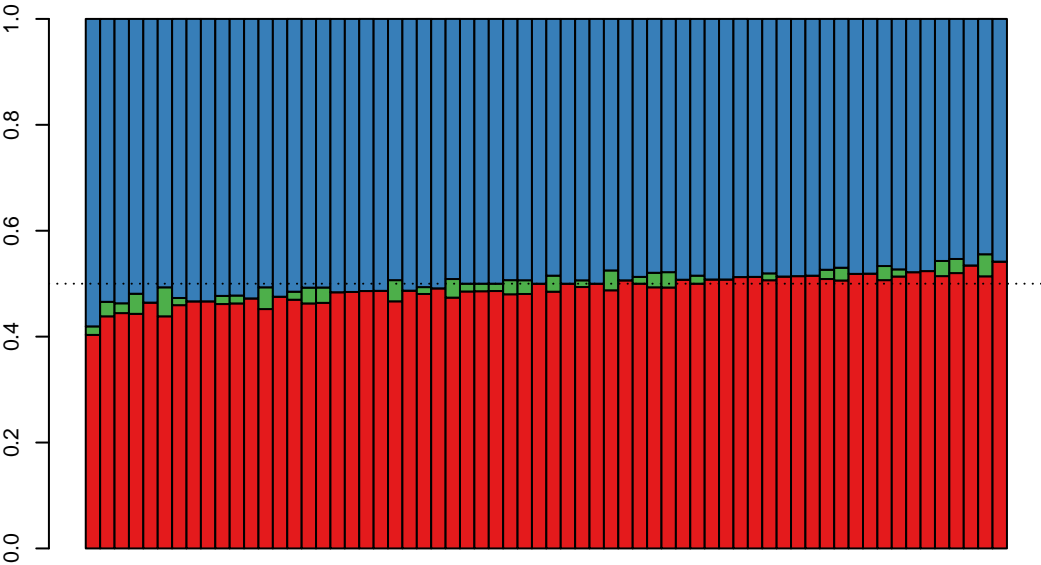

Supplement: Supplementary file 2 [file 3427FigureS2.pdf]

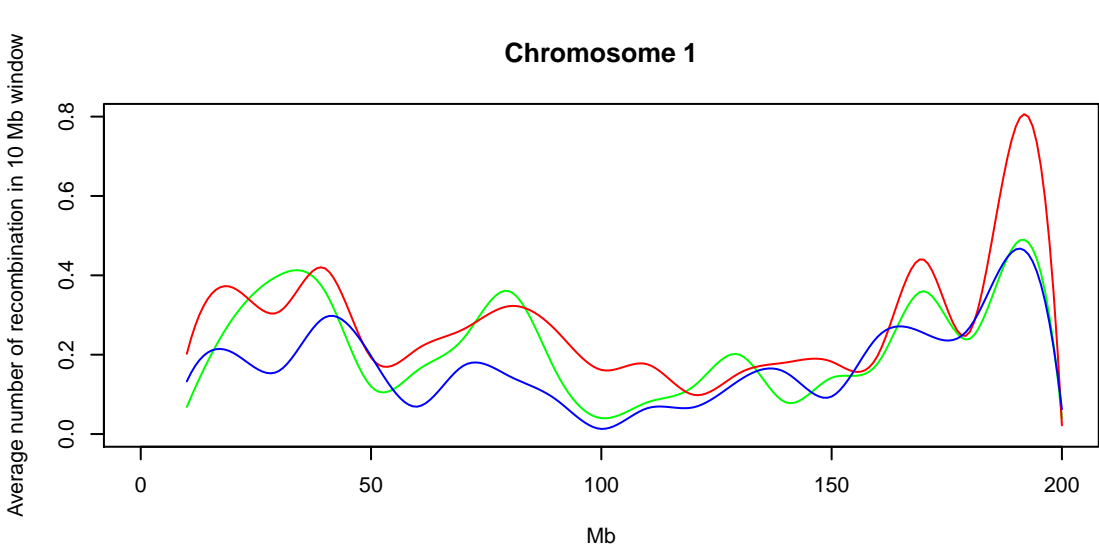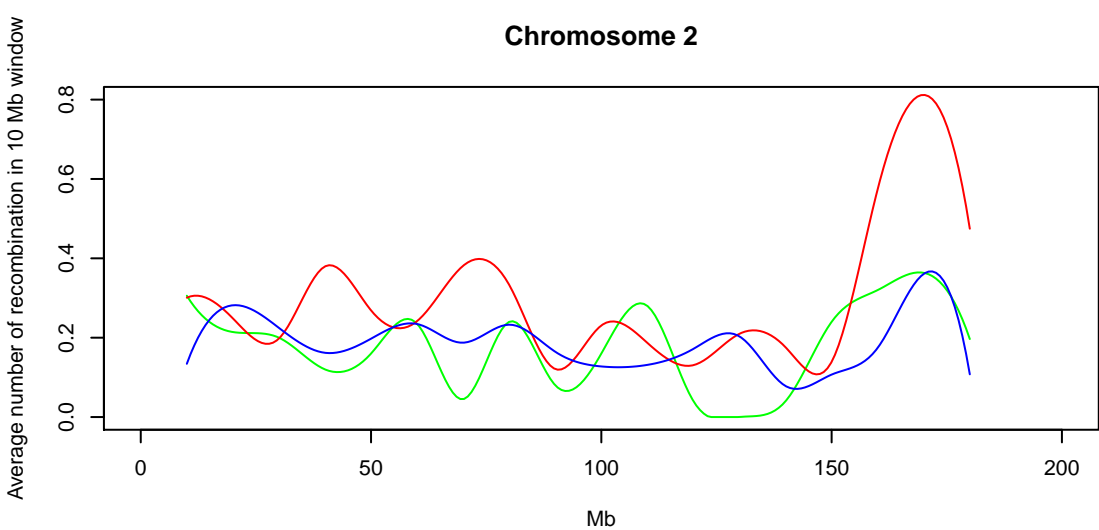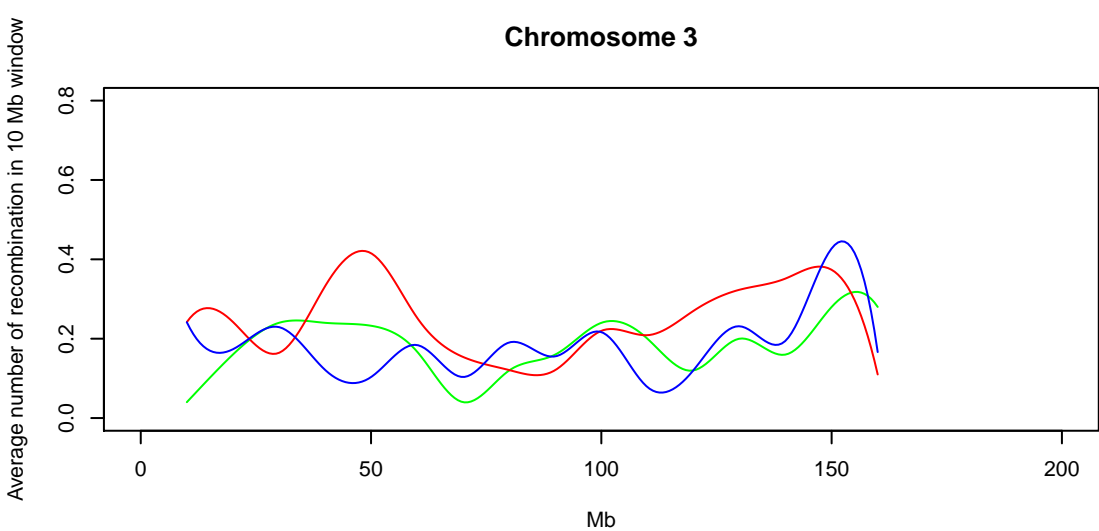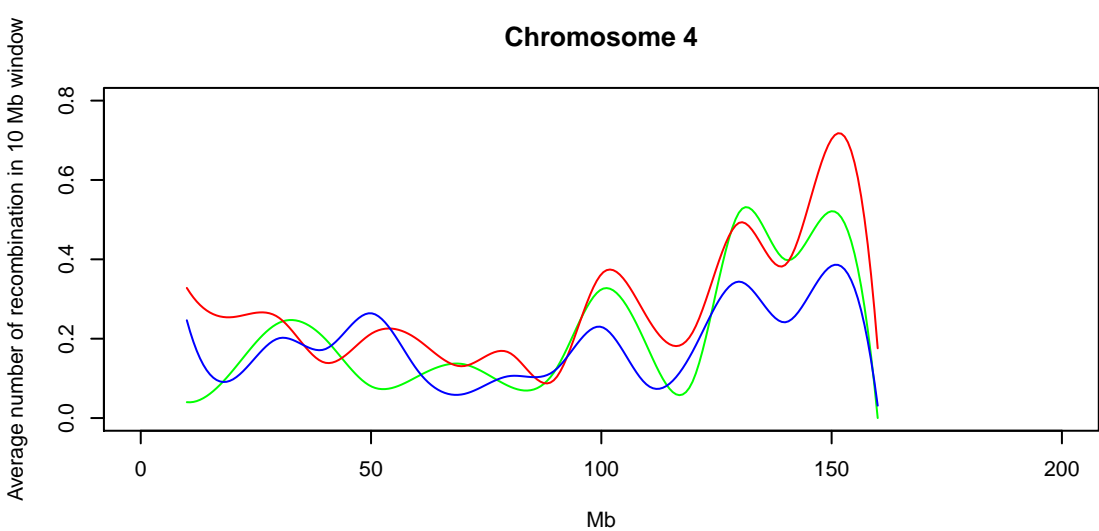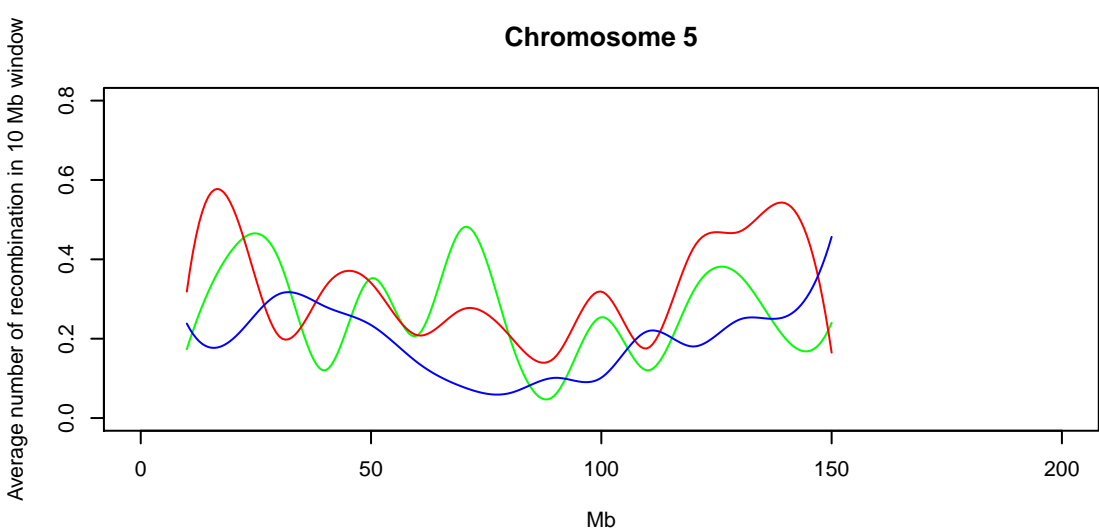

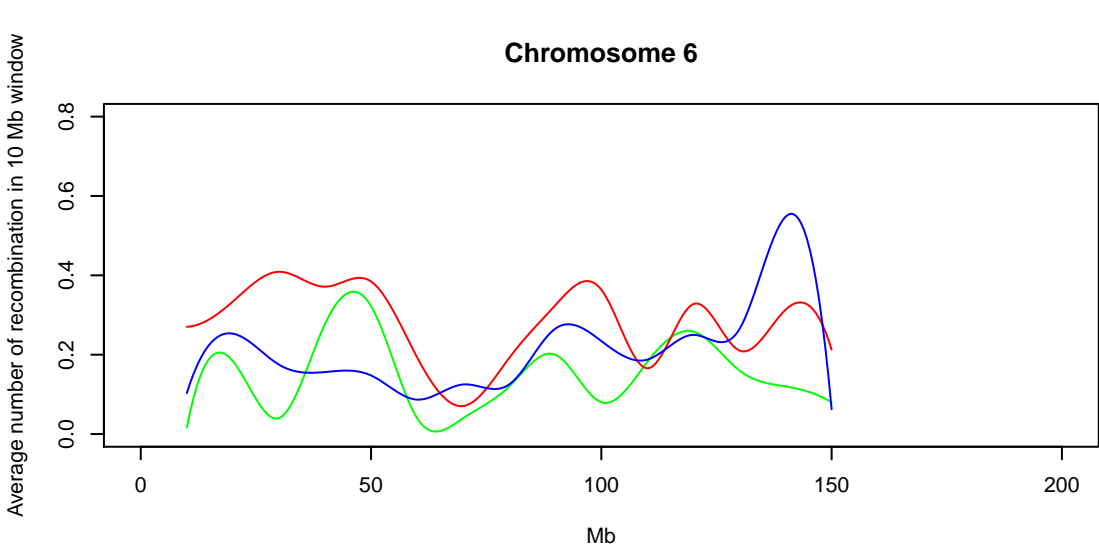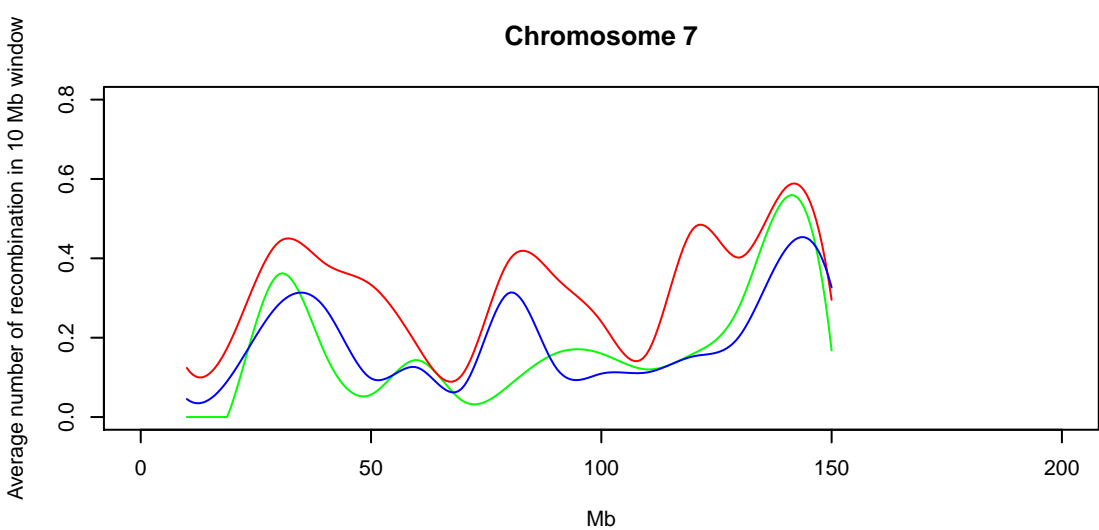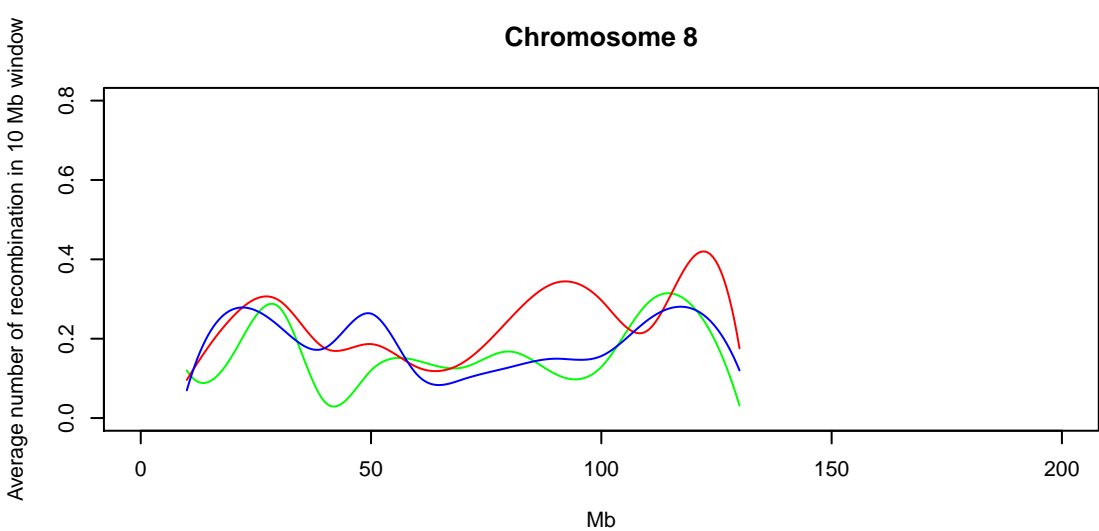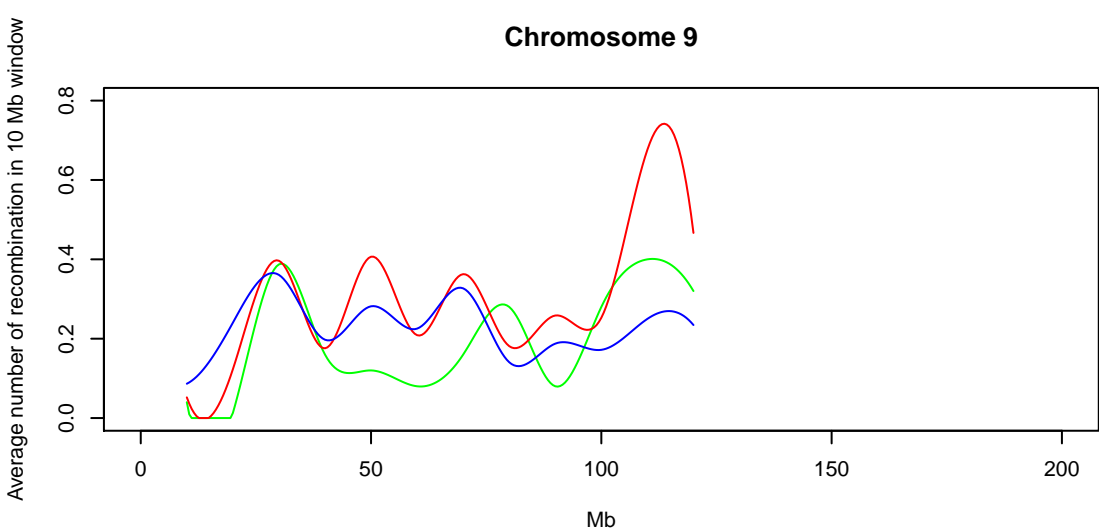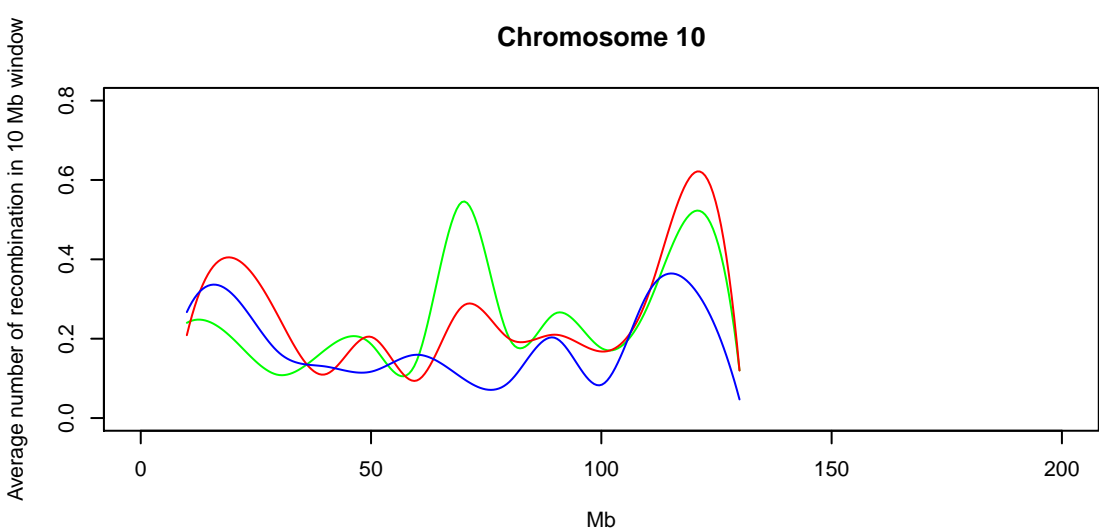

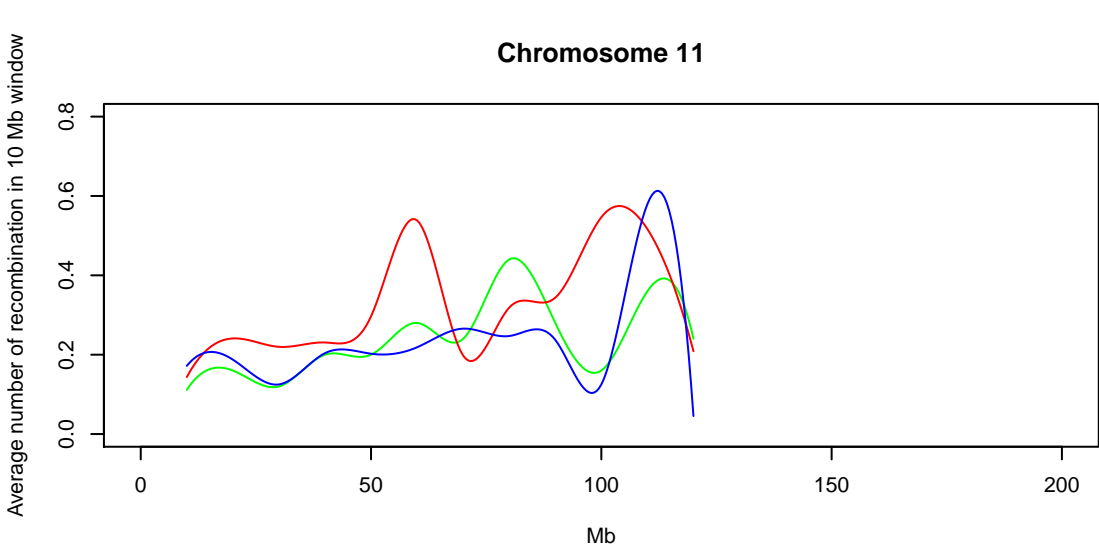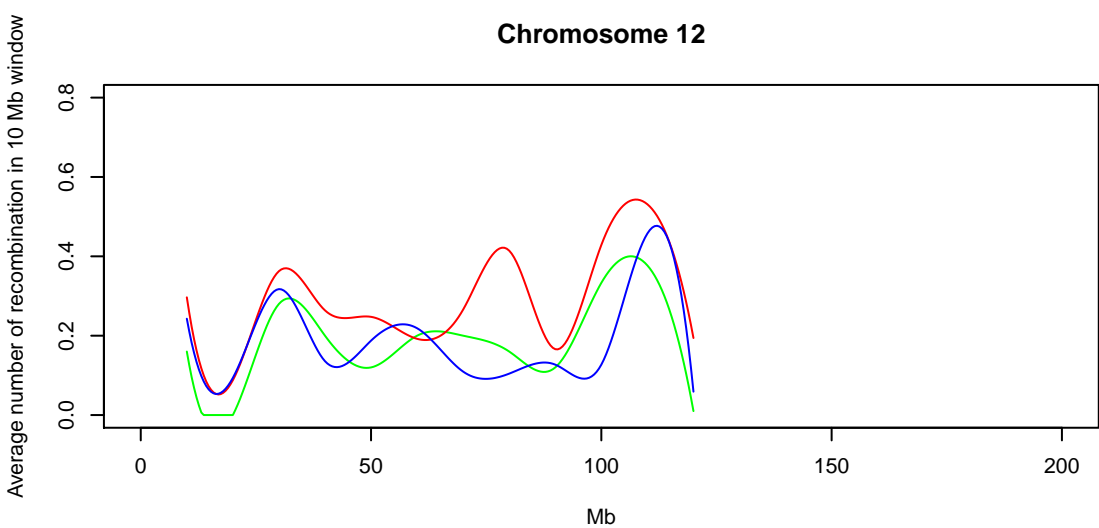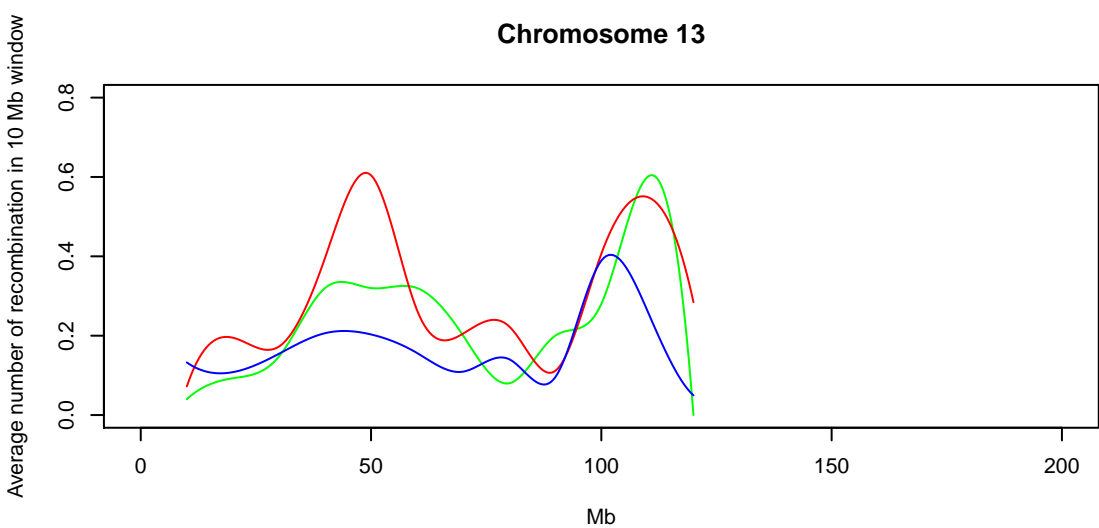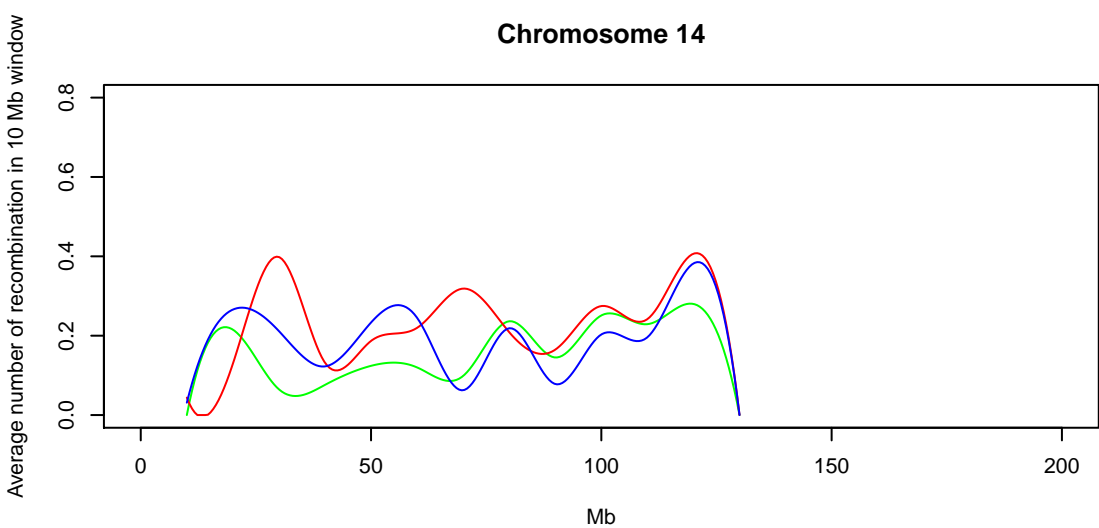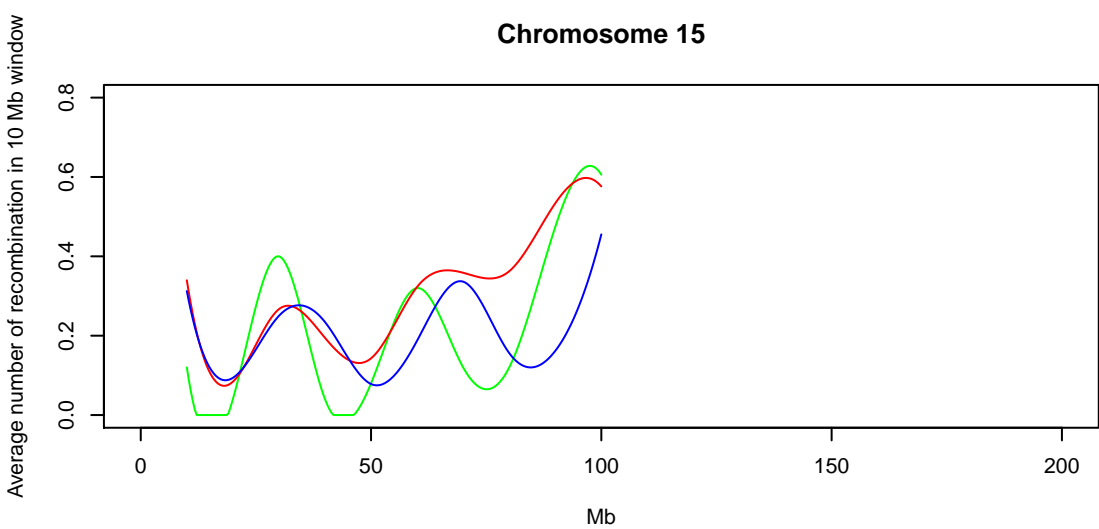

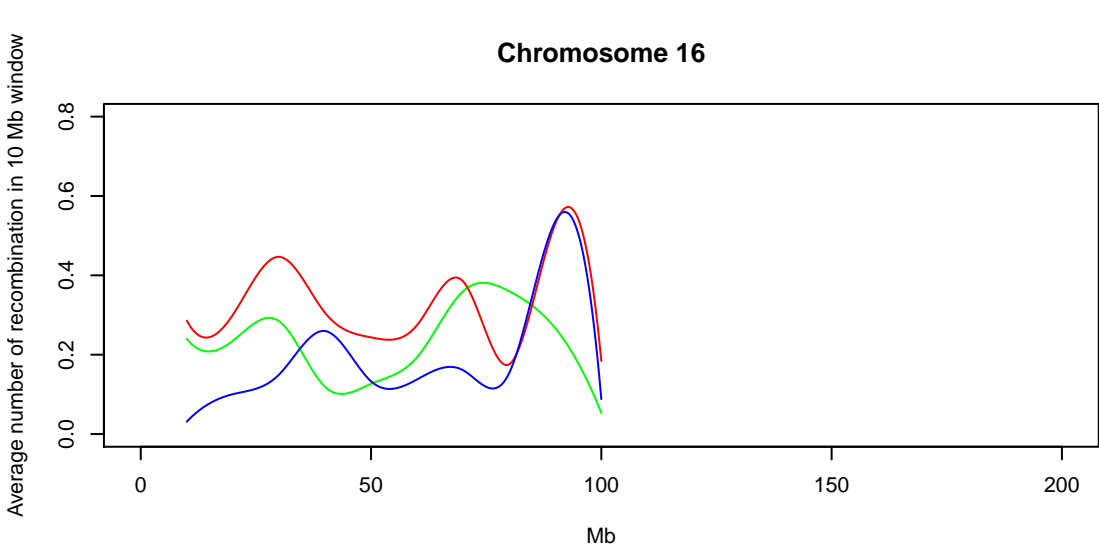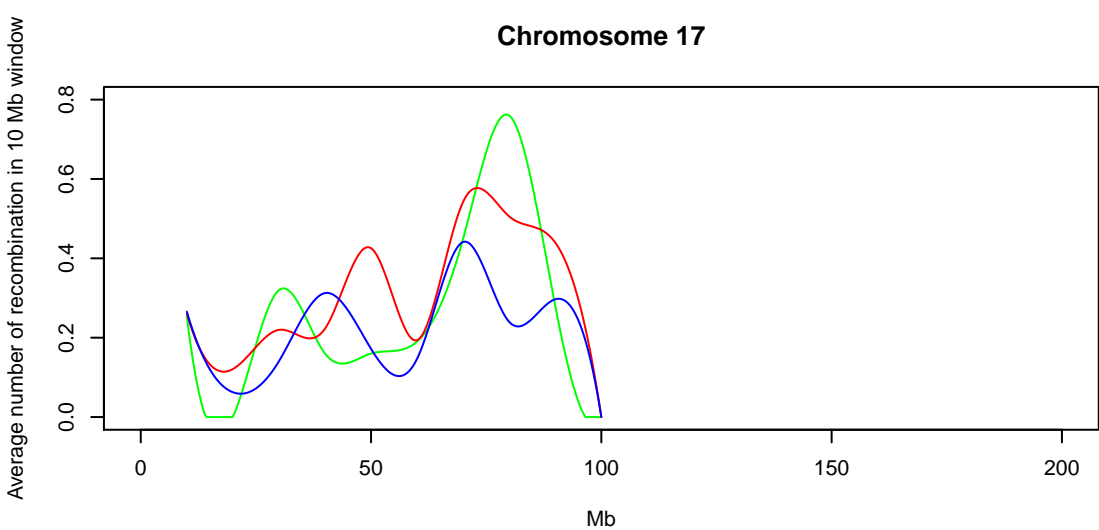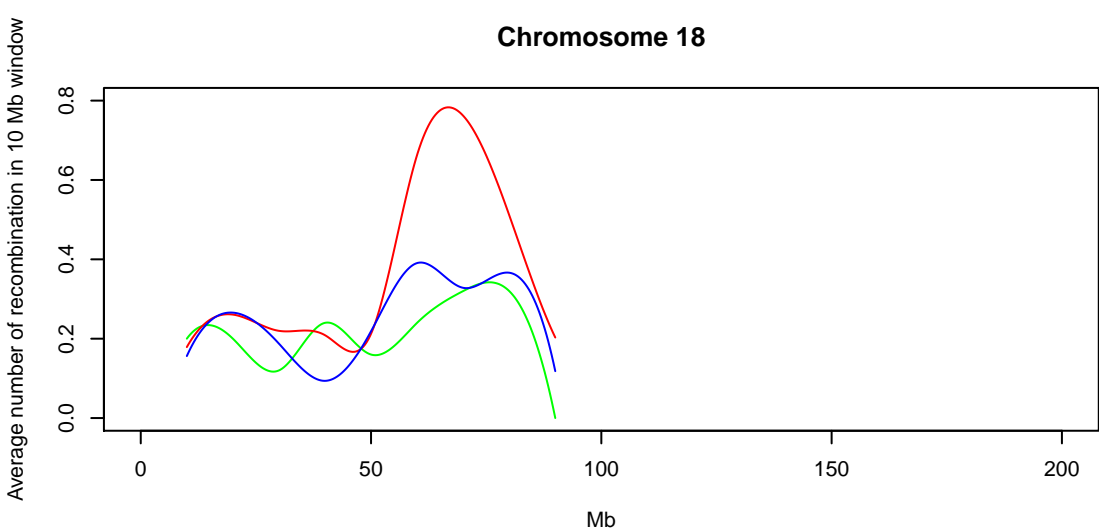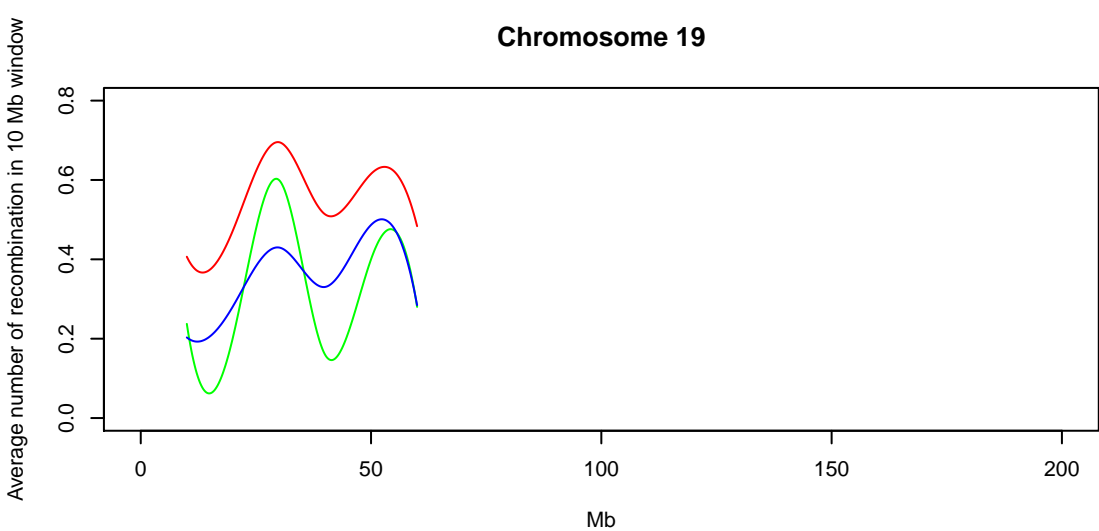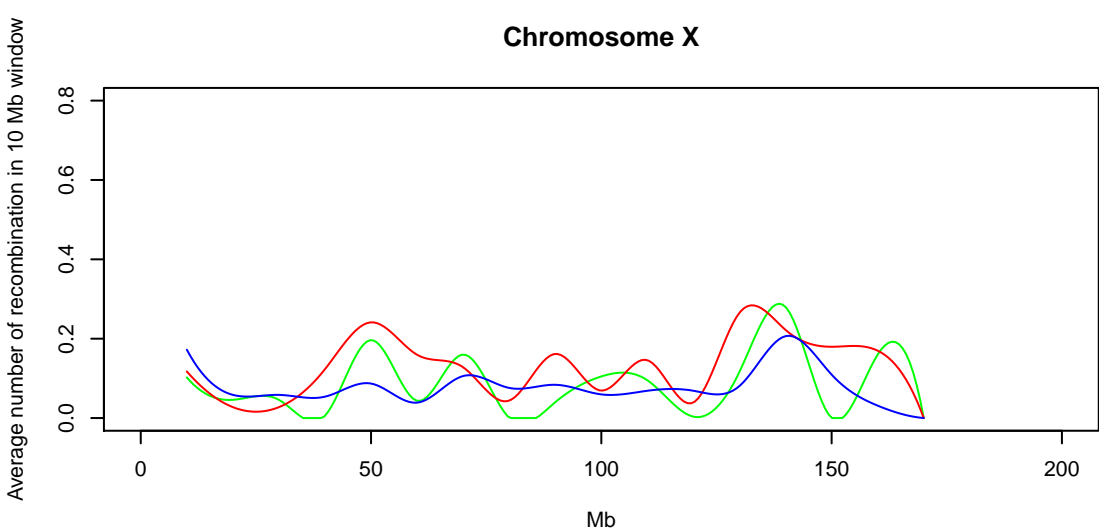

Supplement: Supplementary file 3 [file 3427FigureS3.pdf]
