# Supplementary material for: High-Resolution Maps of Mouse Reference Populations
Source: G3 (Bethesda). 2017 Aug 23;7(10):3427–34. doi: 10.1534/g3.117.300188 (PMC5633391; doi:10.1534/g3.117.300188)
Supplement: Supplementary file 7 [file 3427FileS1.docx]

**Supplemental Figure 1**: Error rate. Each strain is plotted by two dots of different colors (one dot = one founder strain). A dot represents a percentage of markers contradicting the estimated founder strain.

**Supplemental Figure 2**: Percentage of genome attributed to the first or the second RIS founder strain (red = B6 or ILS, blue = A/J or D2 or ISS, green = heterozygous)

**Supplemental Figure 3**: Number of recombinations (smoothed by 10Mb window, AXB = green, BXD = red, LXS = blue).

**Supplemental Table 1**: The list of RIS CNVs (deletion / extra copy).

**Supplemental Table 2**: Number of haplotype blocks (first founder / second founder / heterozygous) and the total number of recombinations.

**Supplemental Table 3**: The list of all recombination intervals (and the frequency of recombination).
